# Induction of Extracellular Aminopeptidase Production by Peptides in Some Marine Bacterial Species

**DOI:** 10.1264/jsme2.ME20150

**Published:** 2021-03-13

**Authors:** Suzune Shindoh, Yumiko Obayashi, Satoru Suzuki

**Affiliations:** 1 Center for Marine Environmental Studies, Ehime University, Matsuyama, Ehime 790–8577 Japan

**Keywords:** marine bacteria, aminopeptidase, starvation, peptide, induction

## Abstract

Bacterial extracellular aminopeptidases are key enzymes in protein processing in oligotrophic seawater. To the best of our knowledge, the regulation of aminopeptidase production in microbes inhabiting seawater has not yet been reported. The present study attempted to experimentally clarify which organic materials affect bacterial extracellular aminopeptidase production by nutrient-rich and starved cells growing in artificial seawater using *Photobacterium*, *Alteromonas*, *Ruegeria*, and *Sulfitobacter*. In all four species, we found that peptides induced bacterial extracellular aminopeptidase production. Amino acids led to cell growth with markedly lower aminopeptidase production by *Photobacterium* and *Sulfitobacter*, but not by *Alteromonas* and *Ruegeria*. These results suggest that the extracellular aminopeptidases of marine bacteria are primarily produced on demand in response to the presence of relevant substrates (peptides) in seawater. Peptidyl substances may be regulatory nutrients for marine bacterial growth in aquatic environments.

Dissolved organic molecules in seawater are important substrates for marine heterotrophic bacteria. The ocean contains a large carbon pool of dissolved organic matter (DOM), with the amount of carbon in DOM estimated to be similar to that in atmospheric CO_2_ (Schimel, 1995). However, significant portions of oceanic DOM pools are recalcitrant to microbial degradation and are only rarely used by heterotrophic microbes (Nagata, 2008). Some of the utilizable dissolved organic compounds in seawater may be characterized as free and combined amino acids and sugars (Amon *et al.*, 2001; Nagata, 2008). The uptake of organic molecules across the bacterial membrane is generally limited to molecules smaller than *ca.* 600 Da (Nikaido and Vaara, 1985). Heterotrophic bacteria in seawater typically take up dissolved organic compounds as small molecules released after the partial digestion of extracellular macromolecules, such as proteins or polysaccharides, by extracellular enzymes (ectoenzymes on the bacterial cell surface or enzymes released into the environment) (Azam and Malfatti, 2007). Other than proteins, polysaccharide fragments may be taken up through Sus-type (particularly with SusC/D) systems via ‘selfish uptake’ (Cuskin *et al.*, 2015); this process occurs in marine waters (Reintjes *et al.*, 2017). In this case, polysaccharides must be initially hydrolyzed outside the cell by extracellular enzymes.

In natural seawater, the activities of proteases have been characterized based on assays that use substrate proxies (Hoppe *et al.*, 2002). Leucine aminopeptidase has been examined as a representative of heterotrophic microbial proteolytic activity in seawater for several decades (Chróst, 1990). Our previous findings suggested that the bacterial utilization of peptidyl compounds in aquatic environments employs a two-step process: trypsin-type endopeptidases catalyze the hydrolysis of high-molecular-weight proteins to oligopeptides, which are subsequently hydrolyzed to amino acids by aminopeptidases (Obayashi and Suzuki, 2005, 2008). Cultured bacterial strains isolated from seawater produced extracellular activities corresponding to several types of aminopeptidases (Bong *et al.*, 2013). These findings suggested that heterotrophic marine bacteria produce extracellular aminopeptidases, which convert peptides in seawater to amino acids that are then taken up by organisms. However, protease activities in marine environments may originate not only from heterotrophic bacteria, but also from protists (Thao *et al.*, 2015). Protease production is regulated by environmental factors, and nitrogen limitations and the presence of phosphate have been shown to induce the production of aminopeptidases (Lazdunski *et al.*, 1975; Ludewig *et al.*, 1987). A role has also been reported for organic matter. Algal proteins in river water appear to induce the production of proteases (Hoch *et al.*, 1996), and glycopeptides have been shown to induce the production of the LonA protease by *Pseudomonas aeruginosa* (Marr *et al.*, 2007). The extracellular proteases of marine bacteria are key enzymes in the marine degradation and processing of DOM, as described above; nevertheless, to the best of our knowledge, there is currently no information on the regulation of extracellular protease production by microbes inhabiting oligotrophic seawater.

We hypothesized that proteinaceous substrates control enzyme activity; bacteria under oligotrophic conditions may respond to organic matter substrates by activating efficient biochemical catabolism. Bacteria have generally developed advanced mechanisms for the regulation of catabolic pathways, as reported by Ben Kahla-Nakbi *et al.* (2007) in marine bacteria. Bacteria (*e.g.*, enteric bacteria) under eutrophic conditions may easily access peptide substances; however, since marine bacteria in seawater are generally subjected to largely oligotrophic conditions, they need to expend energy in order to obtain organic nutrients. To reduce energy expenditure, bacteria in seawater may produce extracellular proteases in an “on-demand” manner, synthesizing enzymes only when the relevant substrates are present in the surrounding water. This hypothesis raises the question of whether organic matter other than peptide substrates also contributes to this proposed regulation of enzyme expression.

In the present study, we attempted to clarify extracellular protease production by heterotrophic marine bacteria exposed to biodegradable DOM in aquatic environments. We considered the following (non-exclusive) possibilities: Proteases are induced by proteinaceous substances (substrates) and some or all of the amino acids that are generated by protein degradation. Additionally, other growth substances, such as polysaccharides, may promote enzyme activity. We also considered that the response observed may differ depending on the physiological conditions of bacterial cells upon encountering potential inducers. Therefore, the present study experimentally investigated the mechanisms by which protease activities are induced in bacteria growing in artificial seawater (ASW) under various physiological conditions. We used four species of heterotrophic bacteria isolated from seawater that were confirmed to produce extracellular proteases before the initiation of the study. The cells of each species were tested under two physiological states depending on their previous nutritional conditions. Specifically, each bacterium was cultured in the presence or absence of sufficient nutrients prior to the initiation of the experiments. Nutrient-rich cultured cells and starved cells were suspended in ASW, and their growth and extracellular protease activity were examined over time.

## Materials and Methods

### Bacteria for experiments

Among *Proteobacteria*, the largest bacterial group in the marine environment (Sunagawa *et al.*, 2015), *Alphaproteobacteria* and *Gammaproteobacteria* are the dominant clades (Hagström *et al.*, 2002; Sunagawa *et al.*, 2015). In the present study, we used two species of *Gammaproteobacteria*, *Photobacterium damselae* subsp. *damselae* strain 04Ya311 and *Alteromonas* sp. strain AIS33, and two species of *Alphaproteobacteria*, *Ruegeria* sp. strain F0CS5 and *Sulfitobacter* sp. strain AIS32. *Photobacterium* was isolated from the surface water in Seto Inland Sea, Kagawa Prefecture, Japan (Neela *et al.*, 2007); *Alteromonas* and *Sulfitobacter* were isolated from the surface water of the Uwa Sea in Ehime Prefecture, Japan (Sato-Takabe *et al.*, 2016); and *Ruegeria* was isolated from the surface water of the Seto Inland Sea, Ehime Prefecture, Japan, as part of the present study. All strains were classified based on their 16S rRNA gene sequences.

The protease activity profiles of these four strains were examined in the logarithmic-growth phase, which revealed that all strains exhibited extracellular aminopeptidase activities, whereas trypsin-type activity hydrolyzing Boc-Phe-Ser-Thr-Arg-MCA was weak (Fig. S1). Other hydrolytic activities against 4-methyl-coumaryl-7-amide (MCA) substrates (Obayashi *et al.*, 2017): nine trypsin types and two chymotrypsin types, were rarely detected.

Therefore, in the present study, extracellular aminopeptidase activity was considered to be an index of proteolytic activity.

### Preparation of cells growing under nutrient-rich and starvation conditions

**Fig. 1.**
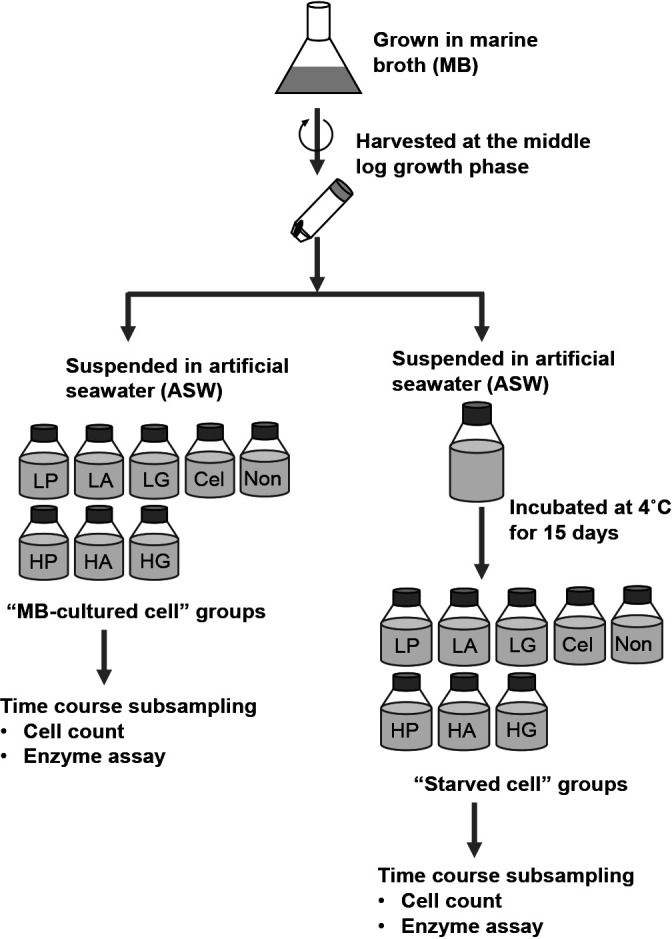
Diagram depicting the experimental design. Details on the organic supplements added to each experimental bottle are described in the text. Abbreviations: HP, High-Peptone; LP, Low-Peptone; HA, High-Amino acids; LA, Low-Amino acids; Cel, Cellulose; HG, High-Glucose; LG, Low-Glucose; Non, without an organic supplement. The experiment was performed in artificial seawater with phosphorus (Fig. 2 and 3) and without phosphorus (Fig. S2 and S3).

The four strains were precultured in 50-mL plastic centrifuge tubes (Sumitomo Bakelite), each containing 20‍ ‍mL of Marine Broth medium (MB; Difco), and incubated at 25°C overnight with shaking at 120 rpm. Cells in the log-growth phase were harvested by centrifugation at 5,400×*g* at 4°C for 30‍ ‍min (Hitachi, CF16RN, T9A31). The resulting pellets were resuspended and washed once with ASW (Table S1), and were then centrifuged again under the same conditions. The resulting pellets were considered to be “cells grown under nutrient-rich conditions” because they had been provided with sufficient organic nutrients, and were hereafter referred to as “MB-cultured cells”. An aliquot of harvested cells was resuspended in ASW and incubated in the absence of any additional organic substances (*i.e.*, under starvation conditions) at 4°C for 15 days. Cells were then collected by centrifugation. The resulting pellets were considered to be “starved cells”. The preparation processes for “MB-cultured cells” and “starved cells” are shown schematically in Fig. 1.

### Microcosms

Aliquots (70‍ ‍mL each) of bacterial cell suspensions in ASW (at 1.0×10^6^‍ ‍cells‍ ‍mL^–1^) were distributed to 100-mL glass screw cap bottles. Separate suspensions were prepared using the MB-cultured cells and starved cells of each strain.

To examine each bacterial growth and their extracellular protease activity in response to the organic matter added, four different organic substances were added to separate bottles (Fig. 1): peptides, amino acids, cellulose (a polysaccharide), and glucose (a monosaccharide), each at two different concentrations (High and Low) (except for cellulose). The peptide supplement consisted of Bacto™ Proteose Peptone No. 3 (BD Bionutrients), which was added to the bottles to a final concentration of 125‍ ‍mg L^–1^ (High-Peptone) or 12.5‍ ‍mg L^–1^ (Low-Peptone). Amino acids were prepared as solutions of 20 types of proteinogenic amino acids (Wako, Nacalai Tesque), which were added to the experimental bottles to a final concentration of 100‍ ‍μM each (High-Amino acids) or 10‍ ‍μM each (Low-Amino acids). Carboxymethyl cellulose sodium salt (CMC, Nacalai Tesque) solution was added to the bottles (Cellulose) to a final concentration of 1.85‍ ‍g L^–1^. D-(+)-glucose (Nacalai Tesque) solution was added to the bottles to a final concentration of 100‍ ‍μM (High-Glucose) or 10‍ ‍μM (Low-Glucose). The experimental concentrations of peptone and CMC were selected based on the expected average molecular weights of each commercial product: 1.25×10^3^ g mol^–1^ for Proteose Peptone and 1.85×10^5^ g mol^–1^ for CMC. Based on these conversions, High-Peptone and Low-Peptone were expected to contain approximately 100‍ ‍μM and 10‍ ‍μM peptone, respectively, while Cellulose was expected to contain approximately 10‍ ‍μM CMC. Due to the lower solubility of polymeric CMC, cellulose supplementation was performed at a single concentration. As a control, a bottle without organic substances (Non) was also prepared. All prepared bottles (High-Peptone, Low-Peptone, High-Amino acids, Low-Amino acids, Cellulose, High-Glucose, Low-Glucose, and Non) were incubated at 25°C without shaking. After 0, 5, 10, 24, 48, and 72 h, 4-mL samples were withdrawn from each bottle and subjected to bacterial counts and enzyme activity assays.

As shown in Table S1, the standard composition of ASW did not include the element phosphorus. To supplement phosphorus in ASW, a 2-mmol L^–1^ phosphate buffer stock was added to ASW to yield a final concentration of 2‍ ‍μmol-P L^–1^, a level that is approximately equivalent to that of total phosphorus in natural seawater (Nausch *et al.*, 2018). Parallel experiments were conducted with and without phosphate-supplemented ASW.

### Viable cell count

Samples were subjected to serial 10-fold dilutions with ASW, and 100‍ ‍μL of diluted samples was plated on each MB agar plate with replicates. After an incubation at 25°C for 1 or 3 days, colonies were counted and viable cell density (colony-forming units [CFU] mL^–1^) was calculated.

### Total cell count

Samples were fixed with glutaraldehyde (0.03% final concentration) at 4°C for 16 or 24 h. Fixed samples then were passed through a 0.2-μm Nuclepore filter (polycarbonate, 25-mm diameter; ADVANTEC) to collect bacterial cells on the filter, and cells were stained with 4,6-diamidino-2-phenylindole (DAPI) as previously described (Bien *et al.*, 2015; Kohyama and Suzuki, 2019). Cell counting was performed using an epifluorescence microscope (×1,000, Olympus BX51) for 30 grids (1 grid: 0.01‍ ‍mm^2^) per sample; the resulting values were used to calculate cell density (cells‍ ‍mL^–1^) in each sample.

### Enzyme activities

Extracellular enzyme activities were measured according to the methods of Obayashi *et al.* (2017). Synthetic MCA substrates (Peptide Institute) were used for the assay: L-leucine MCA (Leu-MCA) and L-alanine MCA (Ala-MCA) are aminopeptidase substrates, and *t*-butyloxycarbonyl-L-leucyl-L-seryl-L-threonyl-L-arginine MCA (Boc-Leu-Ser-Thr-Arg-MCA) is a trypsin substrate. Enzyme activities against these three substrates have been reported in seawater (Obayashi and Suzuki, 2005), and are also produced by marine bacterial isolates (Bong *et al.*, 2013). Working solutions of each MCA substrate were prepared at 2‍ ‍mM in ASW containing 20% dimethyl sulfoxide (Wako). In the assays, 20‍ ‍μL of the MCA substrate solution was mixed with 180‍ ‍μL of each sample in the wells of a 96-well non-binding black plate (Greiner Bio-One) (Obayashi *et al.*, 2017), yielding a final MCA substrate concentration of 200‍ ‍μM. Assays were performed in triplicate (*i.e.*, three wells for each sample). The plate was incubated at 25°C for 1 h, during which time the fluorescence intensity of each well was measured five times (at 12-min intervals) to monitor the time course of the change in fluorescence. The plate was shaken at 800‍ ‍rpm for 60‍ ‍s before each measurement. Measurements were performed using a microplate reader (Corona SH8100Lab) with excitation/emission wavelengths of 380/440‍ ‍nm, respectively. Fluorescence changes were converted to increases in the concentration of a fluorophore (7-amino-4-methylcoumarin; AMC), generated as a hydrolysis product of the MCA substrate. The same analysis was performed with autoclaved samples to assess non-enzymatically produced AMC. The enzymatic hydrolysis rates of substrates in the samples were estimated from changes in the concentration of AMC after the subtraction of non-enzymatically produced AMC. The cell-specific hydrolysis rate (fmol cell^–1^ h^–1^) was calculated by dividing activity by the total cell number at each culture’s sampling time point.

### Statistical analysis

Data are presented as an average±SD where appropriate. A two-tailed one-way ANOVA with post hoc Tukey-Kramer tests were performed to test the significance of differences among the experimental conditions examined. Calculations were performed using Microsoft^®^ Excel^®^ 2016 with a statistical chart for multiple comparisons. A difference was considered to be significant at *P*<0.01 (cell number) or *P*<0.05 (enzyme activity).

## Results and Discussion

### Effects of organic substances on bacterial number

**Fig. 2.**
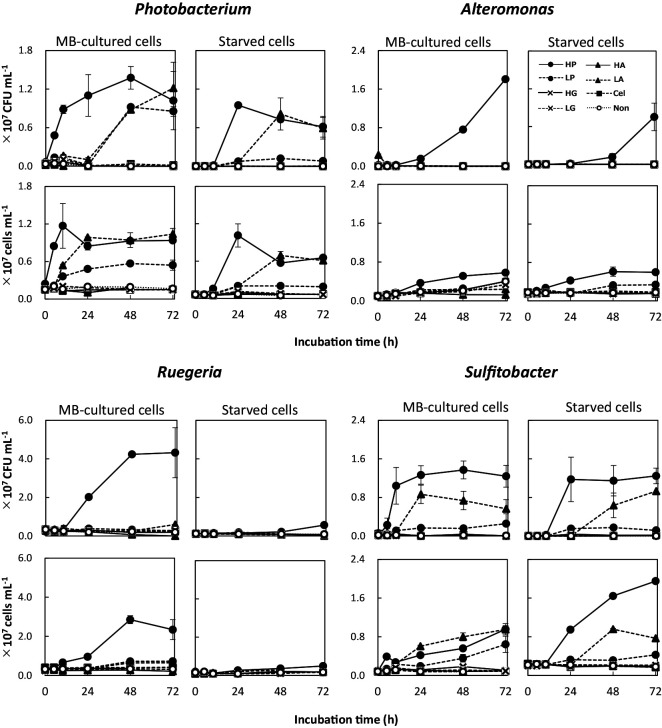
Cell growth of four species in artificial seawater (ASW) supplemented with phosphorus and various organic materials. Cultures were initiated with the MB-cultured cells and starved cells of each bacterial strain. Colony-forming units (CFUs) and total cell numbers were counted at the specified time points. Bacterial strains are indicated with genus names in each graph. Details of added organic materials are provided in the text.

Fig. 2 shows changes in viable and total cell numbers after the separate addition of peptone (peptides), amino acids, cellulose (polysaccharide), and glucose (monosaccharide) to phosphorus-supplemented ASW. In most cases, High-Peptone provided the strongest growth among the experimental conditions examined for both MB-cultured cells and starved cells (Fig. 2). MB-cultured cells of *Photobacterium* and *Sulfitobacter* showed rapid increases in cell density at the early stage (<10 h) of culturing, whereas those of *Alteromonas* and *Ruegeria* increased after 10 h. The lag periods for all species before starting growth were longer with starved cells than with MB-cultured cells, indicating that the resumption of growth in ASW was delayed for starved cells. Growth in High-Peptone was significantly stronger (*P*<0.01) than that in Low-Peptone for all species tested.

When *Photobacterium* and *Sulfitobacter* were cultured in ASW supplemented with phosphorus, growth was observed under not only High- and Low-Peptones, but also Low-Amino acids (Fig. 2). Amino acids were expected to serve as efficient substrates for incorporation and metabolism. Regarding *Alteromonas* and *Ruegeria*, growth was rarely observed in Low-Amino acids and was markedly stronger in peptone-supplemented culture.

Growth was not observed in High-Amino acids medium for any of the tested strains. We postulated that this inhibition was the result of a pH effect: the addition of high concentrations of amino acids lowered the pH of ASW, which was 4.7 in High-Amino acids and 6.0 in Low-Amino acids. The growth pH ranges of each species were previously reported to be 5–11 (optimal pH 7–8) for *Photobacterium* (Moreira *et al.*, 2014), 5–11 (optimal pH 6–8) for *Alteromonas* (Sinha *et al.*, 2017), 5–11 (optimal pH 7–7.5) for *Ruegeria* (Muramatsu *et al.*, 2007), and 6–11 (optimal pH 7–8) for *Sulfitobacter* (Liu *et al.*, 2017). Therefore, the failure of the selected strains to grow in High-Amino acids medium appeared to reflect an inability to grow at low pH.

Carbon, nitrogen, and phosphorus are generally essential for cell growth by bacteria (Vrede *et al.*, 2002). Phosphorus may be a growth-limiting factor for marine bacteria (Cotner *et al.*, 1997). To examine a possible phosphorus effect, we performed the same culturing experiment in ASW with and without phosphorus supplementation. The growth of *Photobacterium* and *Sulfitobacter* was weaker in media lacking supplemental phosphorus (Fig. S2) than in that with supplemental phosphorus (Fig. 2), suggesting that phosphorus facilitates efficient amino acid utilization in the bacteria of these species. A previous study reported that phosphorus limitation negatively affected central metabolism and secondary product synthesis by Gram-positive bacteria (Martín, 2004). However, the physiological effects of phosphorus limitation remain unclear in marine bacteria (Romano *et al.*, 2015), despite marine environments often representing phosphorus-limited conditions. Romano *et al.* (2017) reported a relationship between phosphate limitation and the secretion of ion-chelating molecules by bacteria, which implied that phosphorus deficiency also affected the availability of trace elements. Therefore, the amino acid utilization system may be affected by phosphorus and/or trace metal limitation, leading to growth suppression. The synthesis of nucleic acids may also be involved in the phosphorus limitation effect.

Cellulose and glucose did not induce bacterial growth in any of the strains with or without phosphorus supplementation (Fig. 2 and S2), indicating that saccharides are inadequate as the sole growth substrate. Glucose is a small organic molecule that needs to be readily utilized by bacteria not otherwise limited by a deficiency for an essential element (Labella *et al.*, 2011); in contrast, polysaccharides need to be hydrolyzed prior to the uptake of the resulting saccharides. In the present study, no growth was observed in ASW supplemented with polysaccharides or glucose (at either of the tested concentrations). This impairment in growth was caused by a lack of nitrogen because saccharides do not contain organic nitrogen. ASW does not contain a nitrogen source; therefore, cells cultured in ASW supplemented with either type of saccharide will become nitrogen limited. In contrast, all strains exhibited growth in peptone-supplemented ASW, which is consistent with peptone containing a balance of carbon, nitrogen, and phosphorus.

Therefore, these experiments demonstrated that peptone-supplemented ASW is an efficient growth medium for the bacterial strains tested; ASW supplemented with amino acids also supported the growth of *Photobacterium* and *Sulfitobacter*. In contrast, *Alteromonas* and *Ruegeria* did not show stronger growth in ASW supplemented with amino acids than in ASW lacking any organic substance (Non). Similar results were obtained for the acceleration of growth of starved cells by each organic substance; however, starved cells appeared to require more time to restart their growth in the presence of organic substances.

### Changes in protease activity with each organic substance

Various proteases from marine bacteria have been discovered and examined; however, the majority of studies have focused on proteases as pathogenic factors in fish pathogens (Morita *et al.*, 1996) and as biotechnology tools (Amin, 2018). In ecological studies, proteases have been reported from marine sediment bacteria, a class that primarily includes *Gammaproteobacteria* (Zhang *et al.*, 2015). We herein examined extracellular protease production by four marine strains from *Gamma-* and *Alphaproteobacteria*. We focused on enzyme activities that have been detected in natural seawater (Obayashi and Suzuki, 2005; 2008) and marine bacterial isolates (Bong *et al.*, 2013).

**Fig. 3.**
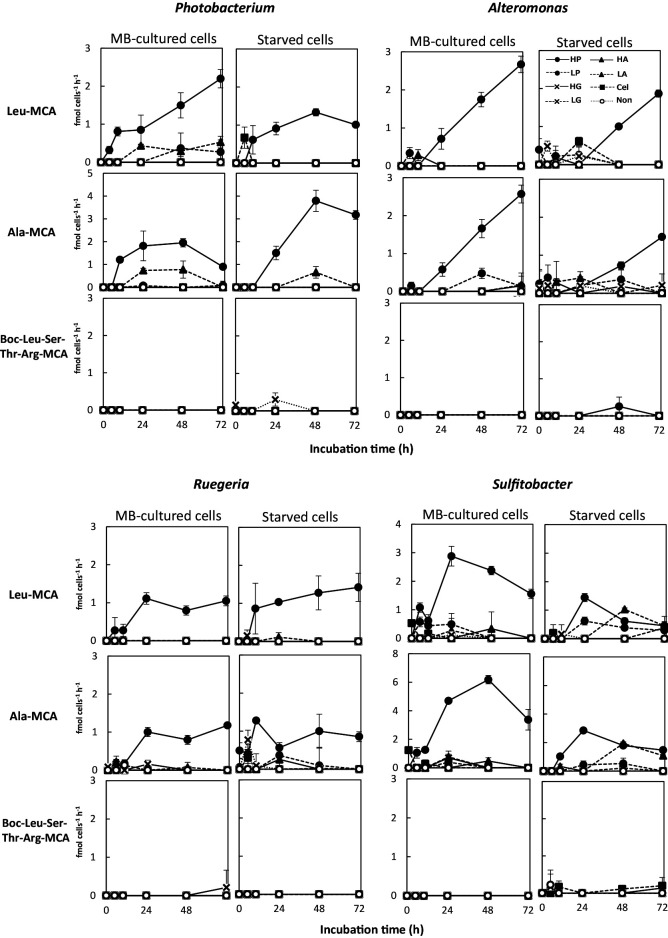
Protease production (cell-specific extracellular activity of the enzyme) by each strain when grown in phosphorus-supplemented artificial seawater (ASW) with various organic materials. Cultures were initiated with the MB-cultured cells and starved cells of each bacterial strain. Enzyme activity was measured for aminopeptidases (with Leu-MCA and Ala-MCA substrates) and trypsin-like enzymes (with Boc-Leu-Ser-Thr-Arg-MCA substrate). Bacterial strains and the symbols for the added organic materials are the same as in Fig. 2.

Fig. 3 and S3 show cell-specific extracellular protease activity during the first 72 h after the addition of substrate organic materials. Cell-specific extracellular aminopeptidase activities (detected by the hydrolysis of Leu-MCA or Ala-MCA) were elevated in some experimental cultures, whereas trypsin-like activities (detected by the hydrolysis of Boc-Leu-Ser-Thr-Arg-MCA) did not exhibit apparent changes from baseline levels in any of the tested experimental conditions (Fig. 3 and S3). Although we cannot completely deny the possibility of the expression of endopeptidases other than trypsin, these strains are not likely to produce endopeptidases because they do not produce them even in MB media (see Materials and Methods).

In experiments performed using phosphorus-supplemented ASW (Fig. 3), the levels of cell-specific aminopeptidase activity were markedly elevated in High-Peptone bottles for both the MB-cultured and starved cells of all tested strains, and were moderately elevated (in some cases) in Low-Peptone and Low-Amino acids bottles. Elevations in the cell-specific activities of enzymes implies enzyme synthesis (enzyme production). Cells cultured in medium supplemented with cellulose or glucose did not show any increases in aminopeptidase activity for any of the strains. Eguchi *et al.* (1996) previously reported that *Sphingomonas* sp. produced proteins and grew in glucose-supplemented ASW medium, which is an oligotrophic condition. The conditions used in the present study lacked a nitrogen source in saccharide-supplemented ASW; therefore, the *de novo* synthesis of enzymes did not occur.

The aminopeptidase activities of all four strains cultured under High-Peptone conditions increased during the logarithmic growth phase (5 to 48 h, depending on the strain, Fig. 2 and 3), and decreased in the stationary phase (after 72‍ ‍h for *Photobacterium* and *Sulfitobacter*). The sole exception was the Leu-MCA hydrolyzing activity of *Photobacterium*, which continued to increase during the stationary phase. *Alteromonas* cultured in High-Peptone continued growing and exhibited increased enzyme activity at 72‍ ‍h (Fig. 2 and 3). When grown in High-Peptone medium, the starved cells of each strain exhibited similar aminopeptidase activity to that of MB-cultured cells. The starved cells of *Ruegeria*, when grown in High-Peptone medium, also exhibited strong aminopeptidase activity before cell growth (Fig. 2 and 3). These results suggest that aminopeptidase production by starved cells was rapidly stimulated upon exposure to peptides, which may be a prerequisite reaction to obtain carbon, nitrogen, and energy for cell growth.

When grown in Low-Amino acids bottles, *Photobacterium* and *Sulfitobacter* also showed aminopeptidase production and cell growth when phosphorus was sufficient (Fig. 2 and 3). Although cell growth by these strains in Low-Amino acids medium was (in some cases) similar to that in High-Peptone medium, aminopeptidase production was markedly lower in Low-Amino acids bottles than that in High-Peptone bottles. Free amino acids, which may be taken up without hydrolysis, are expected to serve well as organic nutrients for heterotrophic bacteria (Azam and Malfatti, 2007). Aminopeptidase activity does not appear to be required for this process.

Bacteria generally encode many types of aminopeptidases. Some of these enzymes are constitutively expressed, while others are induced under specific conditions; however, the regulatory factors that control this expression have not yet been defined for many aminopeptidases (reviewed in Gonzales and Robert-Baudouy, 1996). Phosphorus limitation is considered to be related to the induction of some aminopeptidases located in the cytoplasm or inner membrane of bacteria (Gonzales and Robert-Baudouy, 1996). The results of the present study showed that the extracellular aminopeptidases of the four tested marine bacteria were strongly affected by peptides in the surrounding water, independently of phosphorus limitation. In ecological studies, Hoch *et al.* (1996) reported that aminopeptidases in river microbial communities were induced by proteinaceous substances, a finding that is consistent with the present results. Collectively, these results indicate that a large portion of bacterial extracellular aminopeptidases in aquatic environments are induced in the presence of peptide/proteinaceous substances in environmental water. Marine *Polaribacter* isolates have been reported to possess polysaccharide utilization loci at which peptidase and carbohydrate-active enzymes are coded, forming a chondroitin operon (Xing *et al.*, 2015). This finding suggests that polymer usage is regulated by substrates. The substrate effect has also been reported in carbohydrate enzyme regulation (Koch *et al.*, 2019).

Therefore, extracellular aminopeptidase production by four marine bacterial strains was enhanced in the presence of peptides in the surrounding water. These enzyme activities were induced at the early stages of the growth of these microbes, even in starved cells that did not appear to be proliferating yet. This response to organic substrates is expected to control bacterial growth in oligotrophic aquatic environments.

### Conclusion

The present study examined the growth and extracellular protease activity of four species of *Alpha-* and *Gammaproteobacteria* in the presence of defined medium supplemented with individual organic substances. Growth and increases in cell-specific aminopeptidase activity, namely, aminopeptidase production, were observed for all four tested strains upon supplementation with peptone and amino acids, but not with cellulose and glucose, which may be due to the lack of nitrogen for *de novo* enzyme synthesis. Some of the tested strains showed growth upon supplementation with amino acids with markedly less enzyme production than that with peptone. In some cultures of starved cells, increases in cell-specific aminopeptidase activity appeared before cell growth. These results demonstrate that marine bacteria, whether previously grown under nutrient-rich or starvation conditions, prioritize their response to peptidyl compounds when utilizing nutrients.

## Citation

Shindoh, S., Obayashi, Y., and Suzuki, S. (2021) Induction of Extracellular Aminopeptidase Production by Peptides in Some Marine Bacterial Species. *Microbes Environ ***36**: ME20150.

https://doi.org/10.1264/jsme2.ME20150

## Supplementary Material

Supplementary Material
