# Supplementary material for: Induction of Extracellular Aminopeptidase Production by Peptides in Some Marine Bacterial Species
Source: Microbes Environ. 2021 Mar 13;36(1):ME20150. doi: 10.1264/jsme2.ME20150 (PMC7966946; doi:10.1264/jsme2.ME20150)
Supplement: Supplementary file 1 — Supplementary Material [file 36_20150_s1.pdf]

Supplemental data

Induction of Extracellular Aminopeptidase Production by Peptides in Some Marine Bacterial Species

Suzune Shindoh, Yumiko Obayashi, and Satoru Suzuki

Table S1. Basic composition of artificial seawater.

Component	
NaCl	30.0 g
KCl	0.70 g
$MgSO_4 \cdot 7H_2O$	5.32 g
$MgCl_4 \cdot 6H_2O$	10.7 g
$CaSO_4 \cdot 2H_2O$	1.26 g
(2 mM Phosphate buffer*	1mL)
Distilled water	1.00 L

*Phosphorus is supplemented as phosphate in Figs. 1 and 2

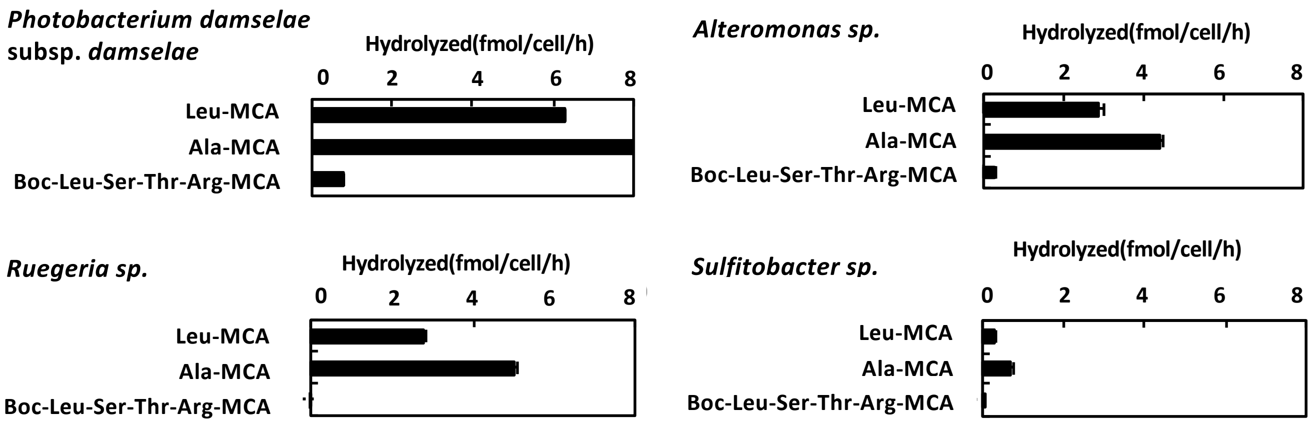

Fig. S1. Protease production profiles of the bacterial strains employed in this study, including: *Photobacterium damselae* subsp. *damselae* 04Ya311, *Alteromonas* sp. AIS33, *Ruegeria* sp. F0CS5 and *Sulfitobacter* sp. AIS32. Representative enzyme substrates consisted of Leu-MCA (aminopeptidase substrate), Ala-MCA (aminopeptidase substrate), and Boc-Leu-Ser-Thr-Arg-MCA (trypsin-like-enzyme substrate).

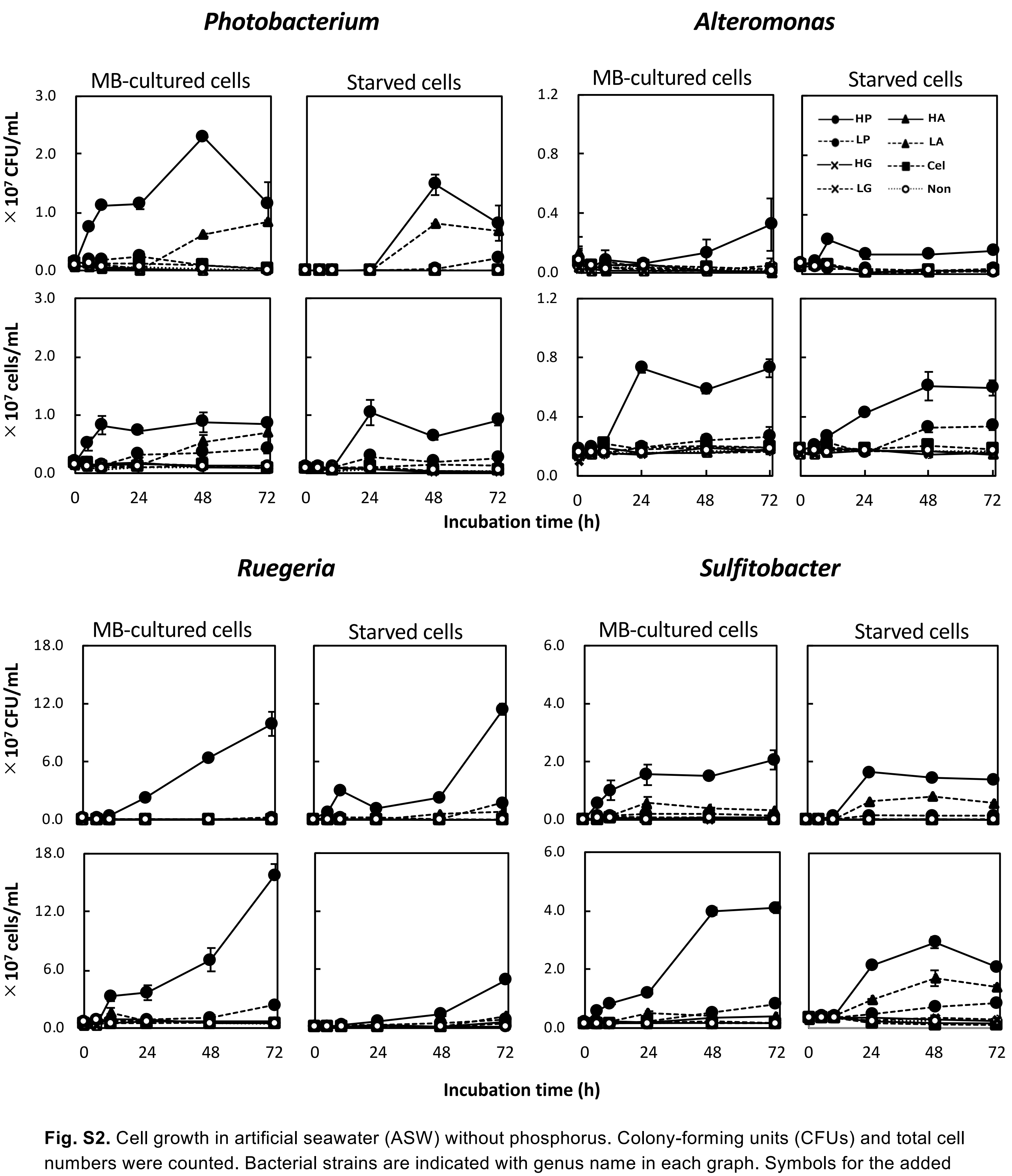

Fig. S2. Cell growth in artificial seawater (ASW) without phosphorus. Colony-forming units (CFUs) and total cell numbers were counted. Bacterial strains are indicated with genus name in each graph. Symbols for the added organic materials are the same as in Fig. 2.

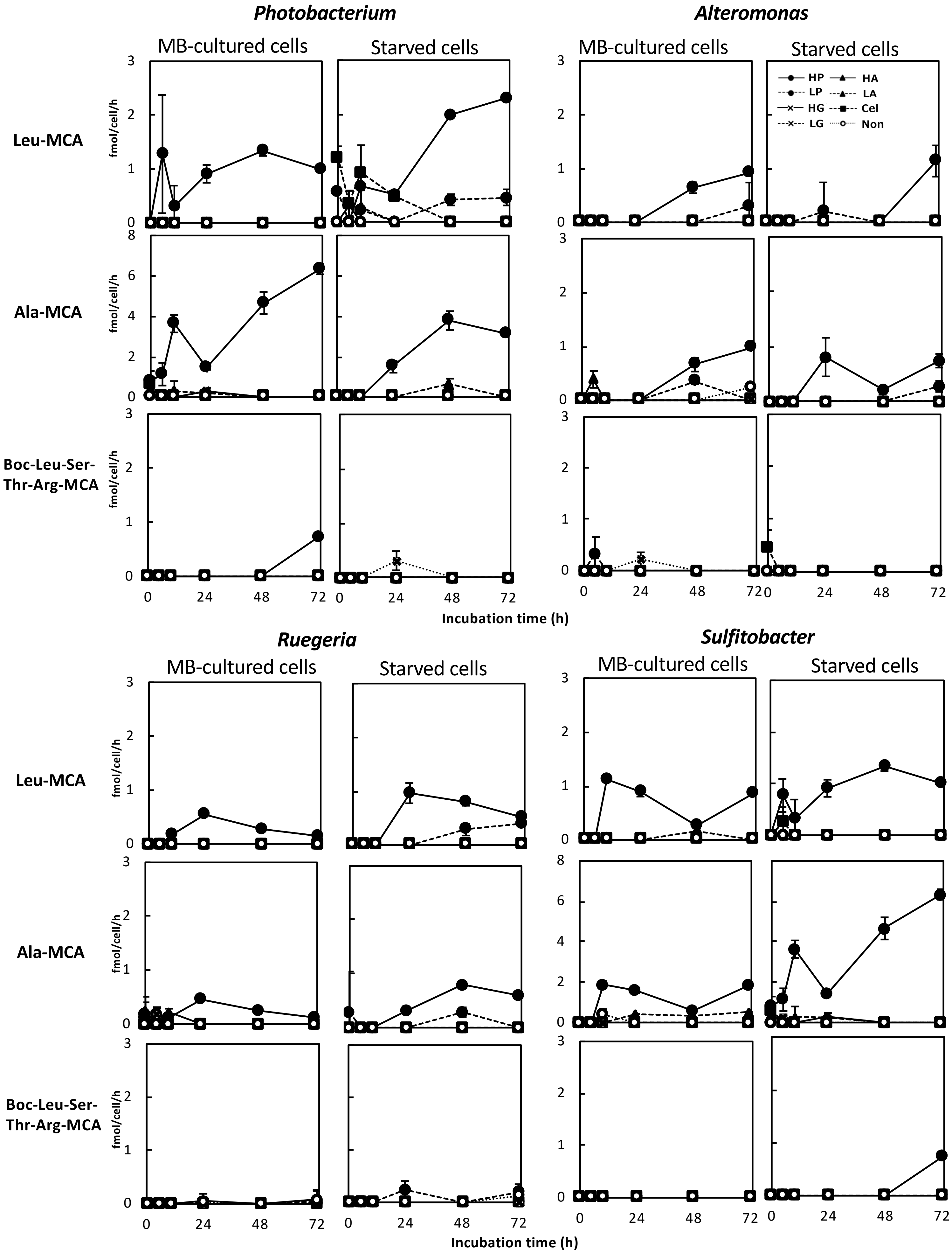

Fig. S3. Protease production (cell-specific extracellular activity) in the experiments performed using artificial seawater (ASW) without phosphorus. Enzyme activity was measured for aminopeptidases (with Leu-MCA and Ala-MCA substrates) and trypsin-like enzymes (with Boc-Leu-Ser-Thr-Arg-MCA substrates). Bacterial strains and symbols for added organic materials are the same as in Fig. 3.
